# Supplementary figures and images for: Pruning deep neural networks generates a sparse, bio-inspired nonlinear controller for insect flight
Source: PLoS Comput Biol. 2022 Sep 27;18(9):e1010512. doi: 10.1371/journal.pcbi.1010512 (PMC9543948; doi:10.1371/journal.pcbi.1010512)

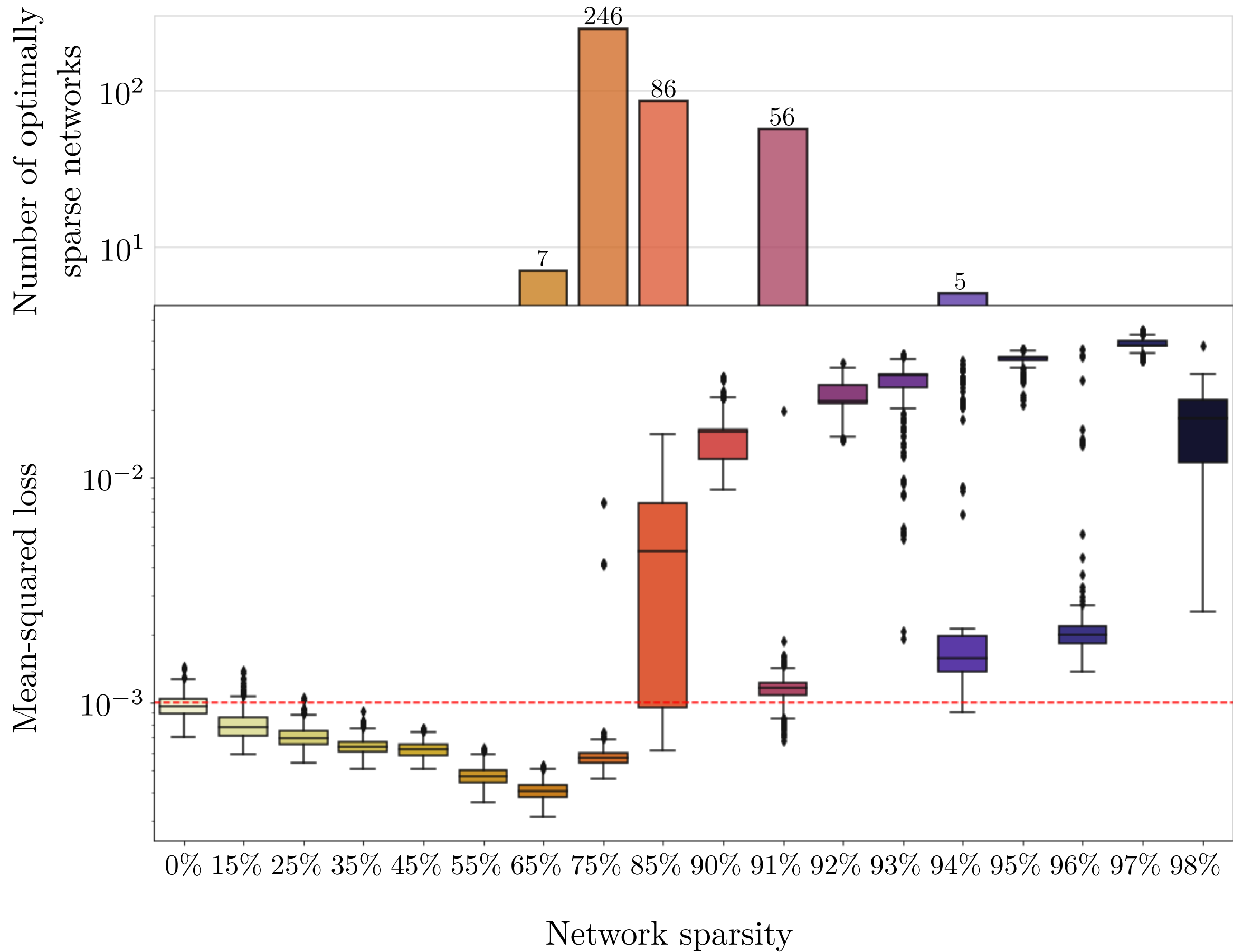

Supplement: S1 Fig — 400 networks, each with four hidden layers with 200, 200, 200, and 8 nodes, respectively, are sequentially pruned and loss of the pruned networks at each sparsity percentage is recorded in the box plot. The bar plot records the number of networks that make it to the corresponding sparsity percentage before exceeding the hypothetical threshold (10−3). (PDF) [file pcbi.1010512.s001.pdf]

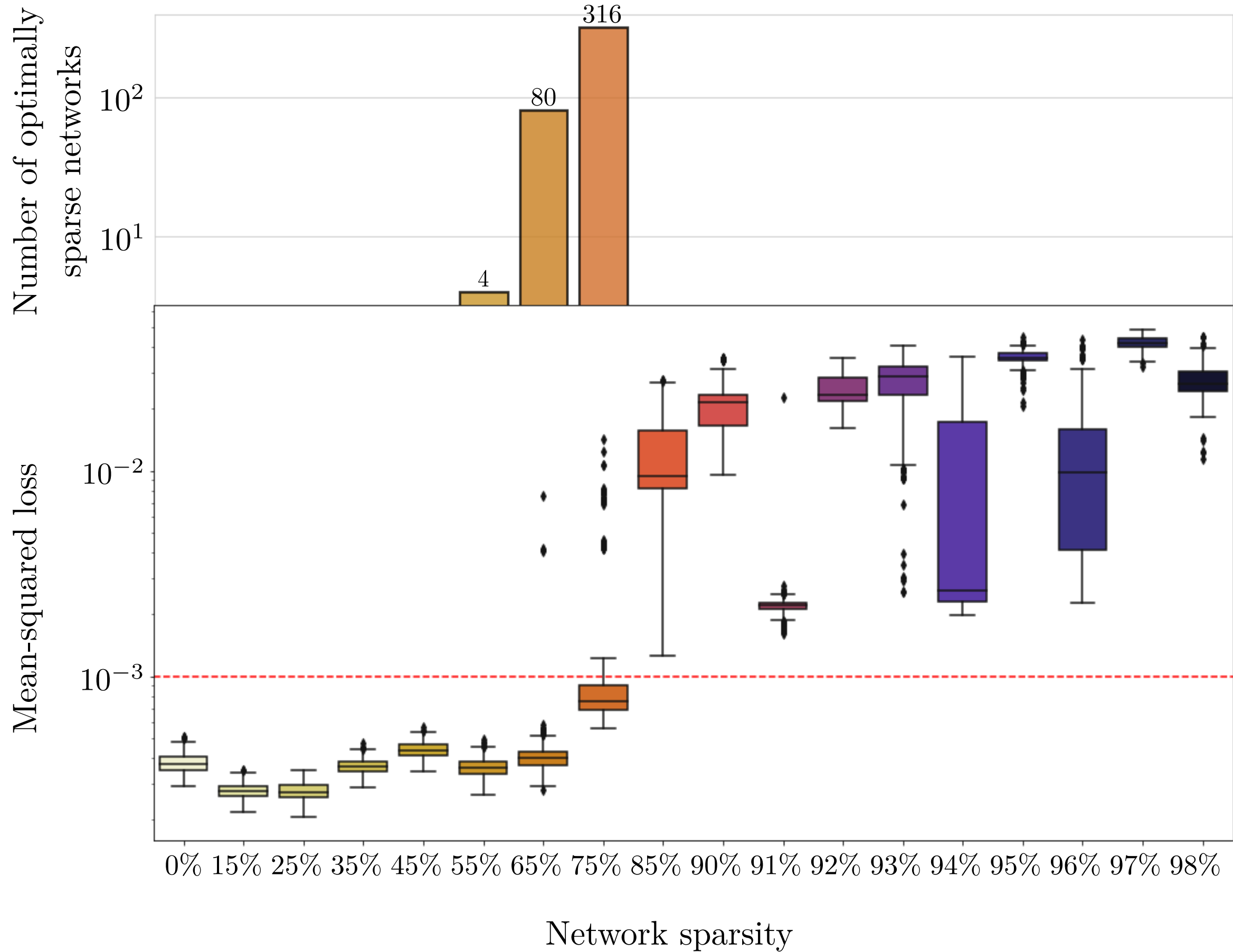

Supplement: S2 Fig — 400 networks, each with four hidden layers with 100, 100, 100, and 8 nodes, respectively, are sequentially pruned and loss of the pruned networks at each sparsity percentage is recorded in the box plot. The bar plot records the number of networks that make it to the corresponding sparsity percentage before exceeding the hypothetical threshold (10−3). (PDF) [file pcbi.1010512.s002.pdf]

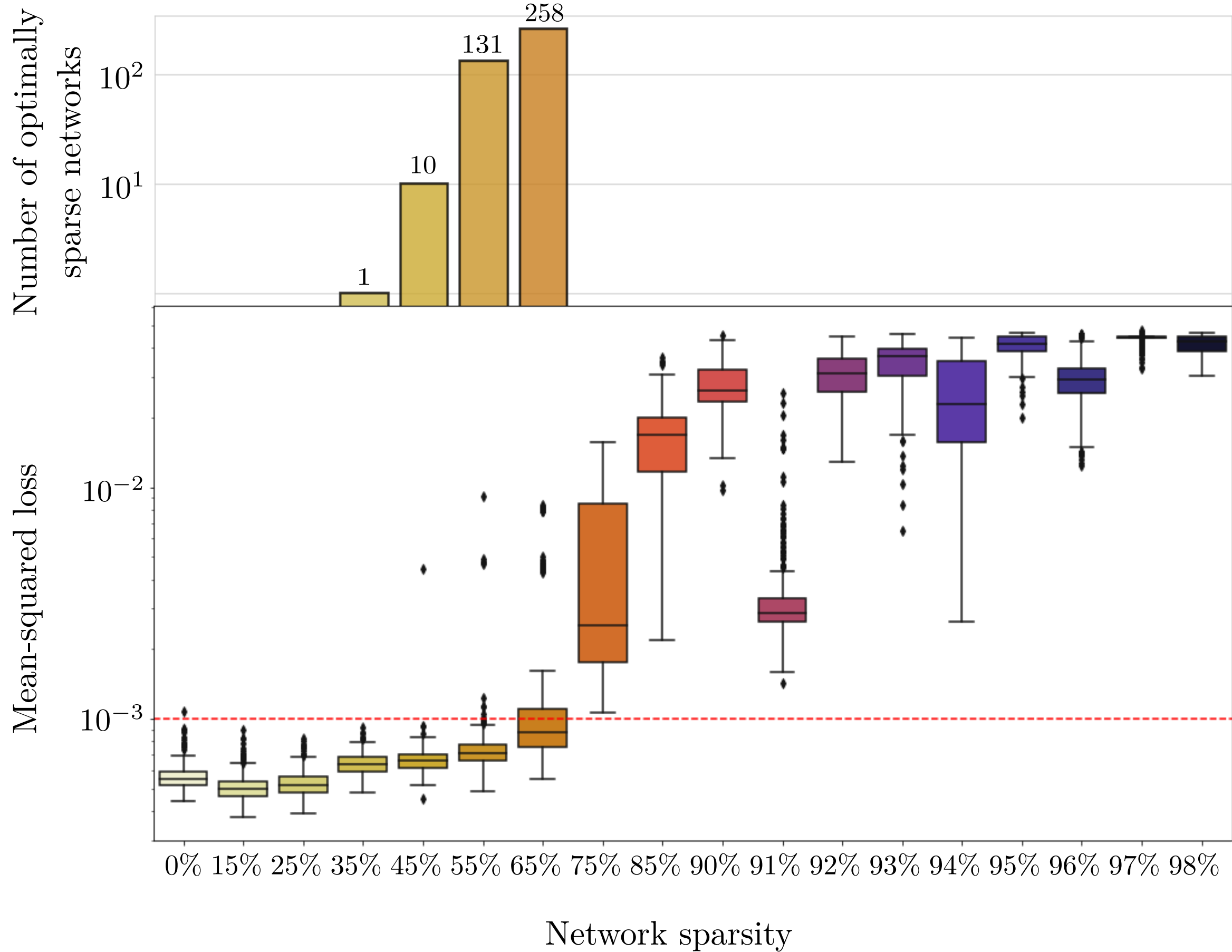

Supplement: S3 Fig — 400 networks, each with four hidden layers with 50, 50, 50, and 8 nodes, respectively, are sequentially pruned and loss of the pruned networks at each sparsity percentage is recorded in the box plot. The bar plot records the number of networks that make it to the corresponding sparsity percentage before exceeding the hypothetical threshold (10−3). (PDF) [file pcbi.1010512.s003.pdf]

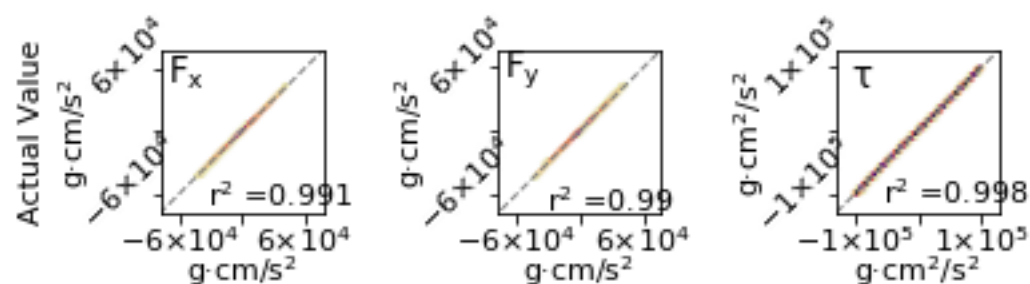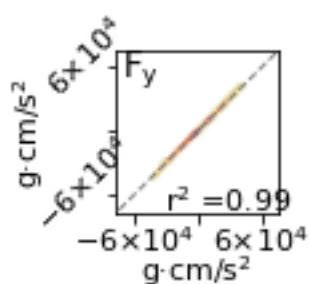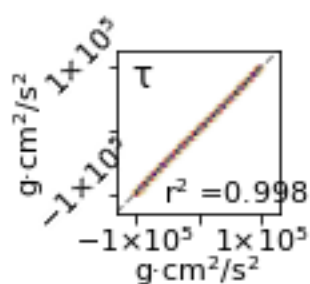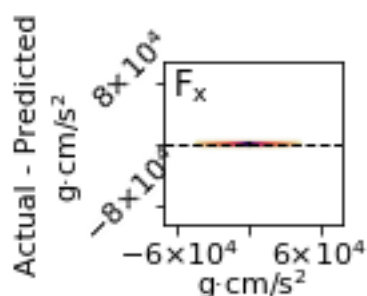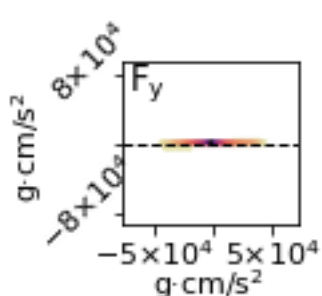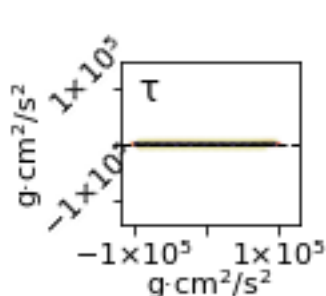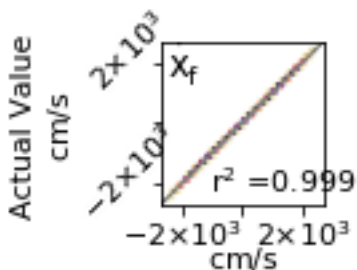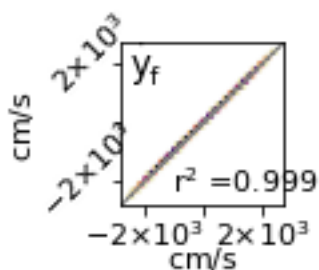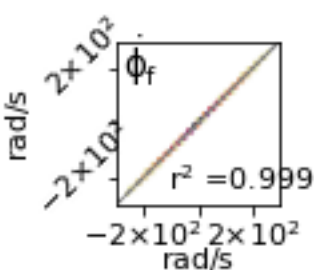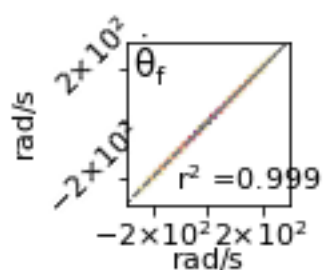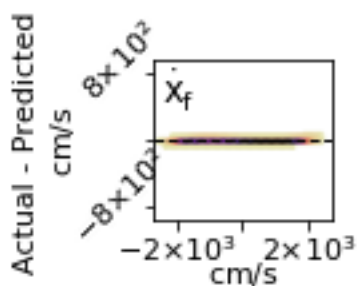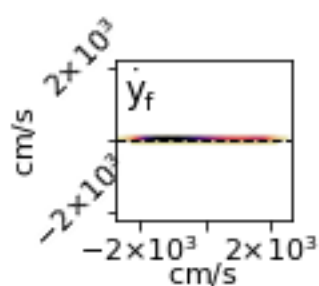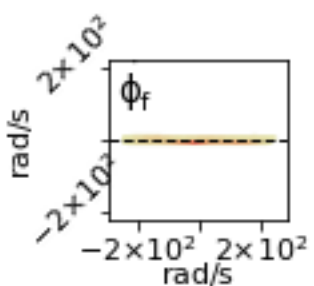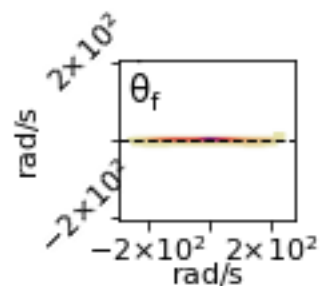

Predicted Value

Supplement: S4 Fig — Error evaluation of a fully-connected network before any pruning. The seven parameters shown are the outputs of the network, the three control variables and the final derivatives of the state space. The residual plots are also shown (denoted by Actual—Prediction). (PDF) [file pcbi.1010512.s004.pdf]

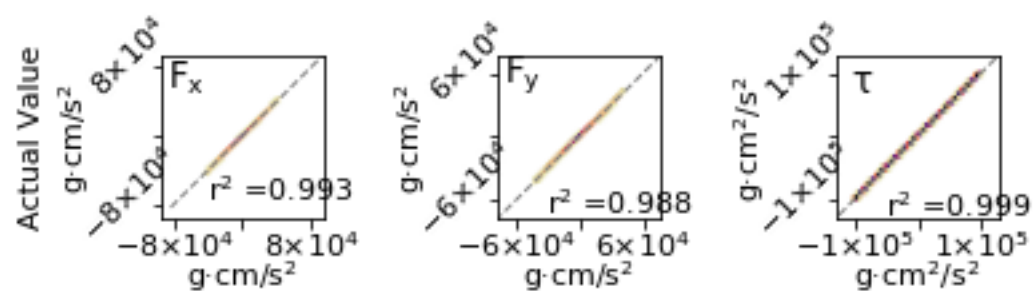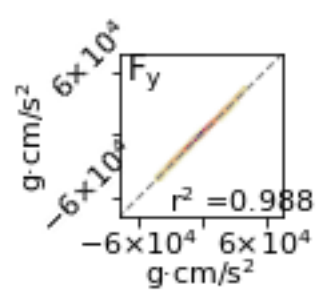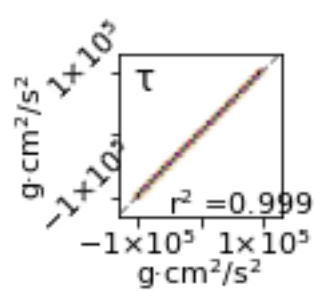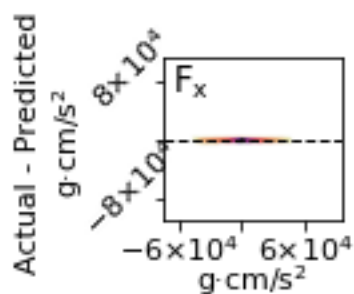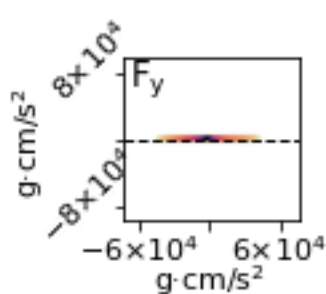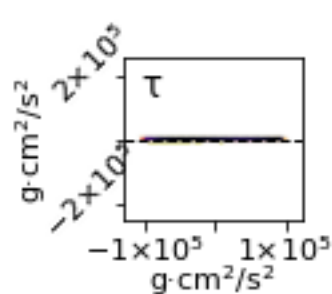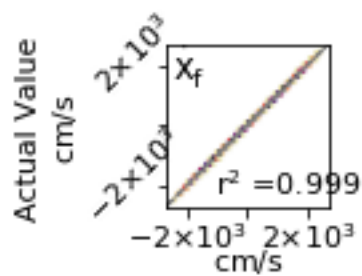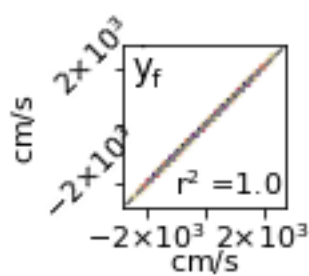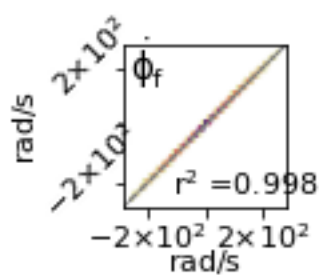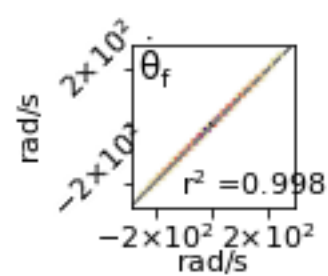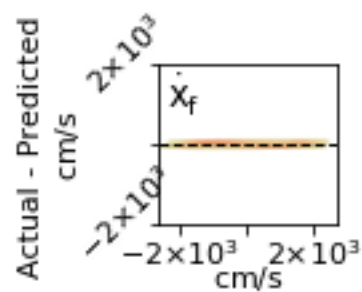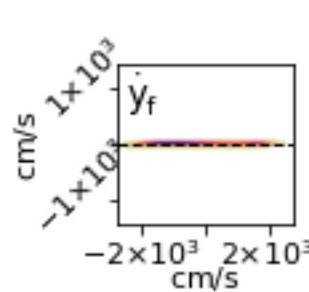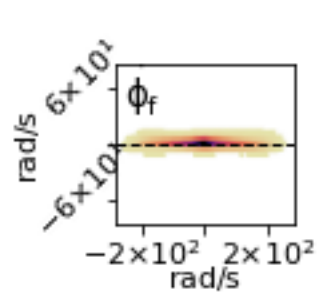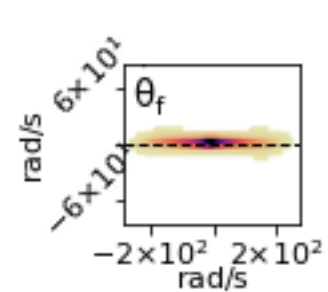

Predicted Value

Supplement: S5 Fig — See S4 Fig. Note that axes for residual plots are scaled to include the max outliers. (PDF) [file pcbi.1010512.s005.pdf]
