# Supplementary material for: Pruning deep neural networks generates a sparse, bio-inspired nonlinear controller for insect flight
Source: PLoS Comput Biol. 2022 Sep 27;18(9):e1010512. doi: 10.1371/journal.pcbi.1010512 (PMC9543948; doi:10.1371/journal.pcbi.1010512)
Supplement: S1 Table — A table of all the required parameters to recreate the simulated model of a moth. (PDF) [file pcbi.1010512.s006.pdf]

**Global parameters for the moth model.** Below, we show a table of all the required parameters to recreate the simulated model of a moth.

| Label | Value | Units | Description |
|---|---|---|---|
| $L1$ | 0.9 | cm | Length from the thorax-abdomen joint to the center of mass of the head-thorax |
| $L2$ | 1.9 | cm | Length from the thorax-abdomen joint to the center of mass of the abdomen |
| $L3$ | 0.75 | cm | Length from the thorax-abdomen joint to the aerodynamic force vector |
| $\rho_{head}$ | 0.9 | g/cm$^2$ | The density of the insect head-thorax |
| $\rho_{butt}$ | 0.4 | g/cm$^2$ | The density of the insect abdomen |
| $\rho_A$ | 0.00118 | g/cm$^3$ | The density of air |
| $\mu_A$ | 0.000186 | g/cm·s | The dynamic viscosity of air at 27°C |
| $a_{head}$ | 0.9 | cm | Length of 1/2 major axis of head-thorax ellipsoid |
| $a_{butt}$ | 1.9 | cm | Length of 1/2 of major axis of abdomen ellipsoid |
| $b_{head}$ | 0.5 | cm | Length of 1/2 of minor axis of head-thorax ellipsoid |
| $b_{butt}$ | 0.75 | cm | Length of 1/2 of minor axis of abdomen ellipsoid |
| $K$ | 23000 | cm$^2$·g/(rad·s$^2$) | Torsional spring constant of the thorax-abdomen joint |
| $c$ | 14075.8 | cm$^2$·g/s | Torsional damping constant of the thorax-abdomen joint |
| $g$ | 14075.8 | cm/s$^2$ | Acceleration due to gravity |
| $\beta_R$ | 0 | rad | Resting configuration of the torsional spring = (initial abdomen angle) - (initial head-thorax angle) - $\pi$ |
| $t$ | 0.02 | s | Time step |
