# Supplementary material for: Pruning deep neural networks generates a sparse, bio-inspired nonlinear controller for insect flight
Source: PLoS Comput Biol. 2022 Sep 27;18(9):e1010512. doi: 10.1371/journal.pcbi.1010512 (PMC9543948; doi:10.1371/journal.pcbi.1010512)
Supplement: S2 Table — A table of all the required calculated variables to recreate the simulated model of a moth. (PDF) [file pcbi.1010512.s007.pdf]

Calculated variables for moth model. Below, we show a table of all the required calculated variables to recreate the simulated model of a moth.

**Variable**	**Expression**	**Units**	**Description**
m_1	$\rho_{head} \cdot \frac{4}{3}\pi \cdot (b_{head})^2 \cdot a_{head}$	g	Mass of head-thorax
m_2	$\rho_{butt} \cdot \frac{4}{3}\pi \cdot (b_{butt})^2 \cdot a_{butt}$	g	Mass of the abdomen
ec_{head}	a_{head}/b_{head}	N/A	Eccentricity of head-thorax
ec_{butt}	a_{butt}/b_{butt}	N/A	Eccentricity of abdomen
I_1	$\frac{1}{5}m_1 \cdot (b_{head})^2 \cdot (1 + (ec_{head})^2)$	g·cm^2	Moment of inertia of the head-thorax
I_2	$\frac{1}{5}m_2 \cdot (b_{butt})^2 \cdot (1 + (ec_{butt})^2)$	g·cm^2	Moment of inertia of the abdomen
S_{head}	$\pi \cdot (b_{head})^2$	cm^2	Surface area of the head-thorax. In this case, it is modeled as a sphere.
S_{butt}	$\pi \cdot (b_{butt})^2$	cm^2	**Surface area of the abdomen. In this case, it is modeled as a sphere.**
Re_{head}	$\rho_A \cdot \sqrt{\dot{x}^2 + \dot{y}^2} \cdot (2 \cdot b_{head})/\mu_A$	N/A	Reynolds number for the head-thorax
Re_{butt}	$\rho_A \cdot \sqrt{\dot{x}^2 + \dot{y}^2} \cdot (2 \cdot b_{butt})/\mu_A$	N/A	Reynolds number for the abdomen
Cd_{head}	$24/\lvert Re_{head}\rvert + 6/(1 + \sqrt{\lvert Re_{head}\rvert}) + 0.4$	N/A	Coefficient of drag for the head-thorax
Cd_{butt}	$24/\lvert Re_{butt}\rvert + 6/(1 + \sqrt{\lvert Re_{butt}\rvert}) + 0.4$	N/A	Coefficient of drag for the abdomen
