# Supplementary material for: Pruning deep neural networks generates a sparse, bio-inspired nonlinear controller for insect flight
Source: PLoS Comput Biol. 2022 Sep 27;18(9):e1010512. doi: 10.1371/journal.pcbi.1010512 (PMC9543948; doi:10.1371/journal.pcbi.1010512)
Supplement: S3 Table — The following variables are the required state space and control variables along with their ranges for random uniform sampling. (PDF) [file pcbi.1010512.s008.pdf]

Initial state space and controls for generating training data. The following variables are the required state space and control variables along with their ranges for random uniform sampling.

Var.	Distribution	Units	Variable type	Description
x_0	0	cm	State Space	Initial horizontal position
$\dot{x}_0$	$Unif(-1500, 1500)$	cm/s	State space	Initial horizontal velocity
y_0	0	cm	State space	Initial vertical position
$\dot{y}_0$	$Unif(-1500, 1500)$	cm/s	State space	Initial vertical velocity
θ_0	$Unif(0, 2\pi)$	*rad*	State space	Initial head-thorax angle
$\dot{\theta}_0$	$Unif(-25, 25)$	*rad*/s	State space	Initial head-thorax angular velocity
ϕ_0	$Unif(0, 2\pi)$	*rad*	State Space	Initial abdomen angle
$\dot{\phi}_0$	$Unif(-25, 25)$	*rad*/s	State space	Initial abdomen angular velocity
F	$Unif(0, 44300)$	g·cm/s^2	Control	Force magnitude
α	$Unif(0, 2\pi)$	*rad*	Control	Force angle
τ	$Unif(-100000, 100000)$	g·cm/s^2· cm	Control	Torque
