# Supplementary material for: Pruning deep neural networks generates a sparse, bio-inspired nonlinear controller for insect flight
Source: PLoS Comput Biol. 2022 Sep 27;18(9):e1010512. doi: 10.1371/journal.pcbi.1010512 (PMC9543948; doi:10.1371/journal.pcbi.1010512)
Supplement: S4 Table — The following variables are the output from the differential equation solver. These variables describe the final state space of the insect after 20ms. (PDF) [file pcbi.1010512.s009.pdf]

Final state space variables The following variables are the output from the differential equation solver. These variables describe the final state space of the insect after 20ms.

**Variable**	**Units**	**Description**
x_f	cm	Initial horizontal position
$\dot{x}_f$	cm/s	Final horizontal velocity
y_f	cm	Initial vertical position
$\dot{y}_f$	cm/s	Initial vertical velocity
θ_f	*rad*	Final head-thorax angle
$\dot{\theta}_f$	*rad*/s	Final head-thorax angular velocity
ϕ_f	*rad*	Final abdomen angle
$\dot{\phi}_f$	*rad*/s	Final abdomen angular velocity
