# Supplementary material for: Pruning deep neural networks generates a sparse, bio-inspired nonlinear controller for insect flight
Source: PLoS Comput Biol. 2022 Sep 27;18(9):e1010512. doi: 10.1371/journal.pcbi.1010512 (PMC9543948; doi:10.1371/journal.pcbi.1010512)
Supplement: Attachment — Submitted filename: response_to_reviewers.pdf [file pcbi.1010512.s010.pdf]

Responses from authors to comments made by the editor and reviews are in green. Comments copied from the editor and reviewers are in black. Responses to the editor, Reviewer 1 and 2 are directly beneath the relevant comment.

The changes to the manuscript are listed at the end of this letter.

Editor:

The Bustamante model is unavailable online for reference. This appears to be a model for the control of abdominal posture during flight. The actual flight mechanics are controlled by a combination of indirect and direct flight muscles operating the wings (Balint & Dickinson, 2001). How do the investigators reconcile these two models?

Thank you for bringing this to our attention. The paper we refer to was under revision for another journal and has now been resubmitted. We have received permission from the journal to post it on BiorXiv. That paper is now available at https://www.biorxiv.org/content/10.1101/2022.06.01.494358v1.

There is an extensive literature on the synaptic networks underlying insect flight. Pearson & Robertson, 1987; Robertson & Pearson, 1983, 1985; Robertson & Reye, 1988, Burrows, 1996. Do any of these models exhibit the backpropagation network motif, characteristic of machine learning and deep learning.

Thank you for raising this issue. To our knowledge, backpropagation has not been shown to occur in biological neural networks. However, we believe DNNs can still serve as an interesting and powerful model for biological neural network processing. DNNs are capable of modeling high-dimensional, complex, nonlinear relationships between inputs and outputs. They are therefore capable of modeling complex biological systems such as vision and motion-control.

The interesting papers you brought up that examine control circuits for locust flight are indeed important contributions surrounding natural neural networks. Indeed, these and other papers from Pearson and colleagues were fundamental contributions to our understanding of central pattern generators (CPGs). That work has led to further studies of CPGs in both invertebrate and vertebrate locomotion. The DNN and MPC approach we use here for the underlying dynamics model (Bustamante et al, 2022) do not consider CPGs. Rather these use state space data (more like a robotics control) as feedback to the model. As such, we did not lean on the CPG literature.

How are off-target contacts withdrawn? Reference 5 does not contain the term pruning. There appear to be two existing proposed mechanisms for “synaptic pruning”. Synaptic Refinement involves the growth cone (Vonhoff & Keshishian,2017) The other mechanism is phagocytic glial cells (Schafer & Stevens, 2013. Which mechanism is being modeled here?

As you correctly point out, biological mechanisms that underlie synaptic pruning (often activity dependent) have a range of processes including a variety of semaphorins, increased GABAergic signalling, changes in dendritic spine density (with some enigmatic mechanisms), and even neuro-immune interactions. These and related mechanisms are explored in a wonderful recent review in

Nature Reviews (Faust et al.) We have revised the manuscript to highlight these issues (**paragraph 1 of the Introduction**). The pruning process we invoke here is a purely computational instantiation of connection elimination. As such we have considered biological systems as inspiration for pruning rather than our computational approach as a model of pruning mechanisms. We thank you for raising this point and have modified our paper to more clearly draw this connection.

Here is the revised text:

"The biological mechanisms that underlie synaptic pruning (often activity dependent) have a range of processes including a variety of semaphorins, increased GABAergic signaling, changes in dendritic spine density (with some enigmatic mechanisms), and even neuro-immune interactions [6]."

Reviewer #1:

I found this an interesting paper, and the central concept of using network pruning to improve the performance of a model insect flight controller certainly seems worthwhile.

Although I am familiar with the theory of deep neural networks, I am not a practitioner myself, so have confined most of my comments below to the aspects related to insect flight dynamics and control.

It will be important to ensure that at least one of the other reviews secured is from a reviewer with specific expertise in deep networks.

Flight dynamics model:

1. The description of the flight dynamics model relies heavily on an unpublished manuscript cited as being in revision at another journal. For example, the authors write (p8): "We used the ordinary differential equations from [27] (See Appendix, Equations 30-33) to generate a dataset for training the deep neural network." As the present submission has no appendix, I assume that this refers to the appendix of Ref. 27. The summary of the model in Fig. 1 treats these ODEs as a black box, and as they are not stated elsewhere in the manuscript, it follows that the work is not described sufficiently well in the manuscript to be reproducible in its current form. I did try reviewing the supporting GitHub briefly to look for the equations, but don't feel that I should have to go to this trouble or need to read Python to understand the modelling.

We apologize for not having included the manuscript in our original submission to PLoS. The paper was under review and has since been resubmitted and also uploaded to BioRXiv. The link is now indicated in the revised manuscript as well as here. https://www.biorxiv.org/content/10.1101/2022.06.01.494358v1.

2. Taking the model at face value, some further justification and explanation is necessary to demonstrate that it really does provide a meaningful model of insect flight dynamics. Although the limitations of the model are mentioned in general terms on p12, there are several specific features of the model that seem surprising and that would therefore benefit from further justification and explanation.

1. The dynamics of flight result from gravitational and aerodynamic forces, but the summary in Fig. 1 only shows one external force “F”. Gravitational acceleration is listed as a model parameter in Table 2, so I assume that “F” refers to the aerodynamic force only, and that the insect’s body weight is included separately in the model. (If this were not so, such that “F” was in fact the total net force, then I would have other concerns on the constraints imposed by the modelling.) Either way, the fact that there is any uncertainty on this point at all reinforces the need to improve a full explanation of the model, to avoid the reader having to trawl through code to find out. Providing the ODEs is also important to understanding why the network inputs and outputs are set up as they are. Besides providing all relevant equations, I would strongly suggest showing all the relevant forces and torques in Fig. 1 for clarity.

Thank you for raising this point. F is the average force applied by the wings during each downstroke and upstroke. The actual model, now fully available (see above) includes gravitational forces, abdominal torques, drag forces on the body as well as the downstroke or upstroke averaged forces. We have added a comment about this in p**aragraph 2 of the Materials and methods section** in the revised manuscript. We apologize for having not placed the model on BioRxiv sooner.

Here is the revised text:

“... the average force applied by the wings during each downstroke and upstroke; …”

“In addition to the downstroke or upstroke averaged forces, the model includes gravitational forces, abdominal torques, and drag forces on the body.”

2. Under the model, the force F can act in any direction with respect to the axes of the head-thorax element, as its angle alpha is drawn from a uniform distribution on the interval (0, 2pi). This seems implausible, and leaves me feeling that the flight dynamics model is better viewed as a model of a two-body system with thrust vectoring, rather than as a model of insect flight per se. Related to this, it seems from the parameterization that the force F is unaffected by the insect’s body motion, which runs contrary to the results or assumptions of most other insect flight dynamics modelling.

The reviewer is absolutely correct. The model really is a simplified two-body dynamical system with thrust vectoring. And, since our dominant focus was either on hovering flight or on small amplitude body motions, we felt that a simple vectoring analysis was a good starting point. But, as the reviewer correctly pointed out, we did not make this point clear in the manuscript. We have now added a comment to this effect in **paragraph 2 of the Materials and methods section.**

Here is the revised text:

“Thus our model is basically a simplified two-body dynamical system with thrust vectoring. Since our dominant focus is on hovering flight, this provides a reasonable basis for examining the control consequences of pruning a deep neural network.”

3. This being so, it would be reassuring to see some validation of the model. Again, I presume that this is provided by Ref. 27, but as things stand the reader is left without either a principled derivation or an empirical validation to go on. On this basis, although it is clear that the authors have constructed a pruned network architecture that is capable of controlling the motor system that they have modelled, it is less clear what this has to do with insect flight.

Thanks again, as we mentioned above, our intention here was more to use the biological concept of network pruning to control a simple dynamical system. In this instance, our focus was on an inverse problem of a simple insect flight system. We have clarified this issue in **paragraph 2 of the Materials and methods section** in the manuscript (please see the previous revision to the manuscript).

I trust that these comments will be reasonably straightforward to address, but would like to see some more detailed explanation, justification, and validation of the flight dynamics model before making any final recommendation.

Network pruning:

The authors conclude (p13) that "For the task of moth hovering, a DNN can be pruned to approximately 7% of its original network weights and still perform comparably to the fully-connected network." Although the dimensions of the input and output layers are uniquely defined by the model of moth hovering that the authors have used, it appears that the authors have made an arbitrary choice to include 400 nodes in three hidden layers, and 16 nodes in another. I would assume that the quantitative conclusions on pruning rest heavily on this choice, so would like to see some discussion or exploration of this point.

Thank you for this interesting and relevant suggestion. We have performed additional experiments to begin to explore the relationship between initial network size and pruning statistics. The results of those experiments are shown in Supporting Figures 1-3 and discussed in **paragraph 8 of the Discussion section**.

Indeed, the choice of 400, 400, 400, and 16 for the hidden layer widths is somewhat arbitrarily large. However there are a few key reasons why we chose these hyperparameters initially:

1. Through the process of tuning and cross-validating the fully connected network, we converged to a set of hyperparameters (including the size of the hidden layers) which resulted in the most optimally performing network. As is standard in the field of machine learning, we tried different combinations of hyperparameters until we found a set that performed well.
2. Additionally, we wanted the fully-connected network to be highly over-parameterized for the task in order to demonstrate the effects of said over-parameterization and pruning. We found that for the task of insect flight control the network is in fact highly over-parameterized (only 7% of network weights are required for the network to perform below loss threshold).

However, the results of the original study pose an interesting question about how the size of the initial network architecture affects the resultant pruning statistics (as Reviewer #1 alluded to). To begin to

explore the effect that initial network architecture size has on the pruning statistics, we repeated the experiment with increasingly smaller network architectures. Precisely, we repeated the experiments on 3 different feed-forward network architectures:

1. 400 networks with 200, 200, 200, 8 nodes in each hidden layer,
2. 400 networks with 100, 100, 100, 8 nodes in each hidden layer, and
3. 400 networks with 50, 50, 50, 8 nodes in each hidden layer.

The results of these experiments are shown in Supporting Figures 1-3, which are of the same format as Fig. 4 in the main body of the paper. We believe these experiments are primlimary explorations, as they open interesting follow-up questions which we have outlined **paragraph 8 of the Discussion section** of the revised paper.

Here is the revised text:

"The results of this study pose an interesting question about how the size of the initial network architecture affects the resultant pruning statistics. The networks pruned in this study are feed-forward, each with four hidden layers with 400, 400, 400, 16 nodes… As stated, these preliminary experiments open up many interesting questions to be explored in future work."

Reviewer #2:

This is a fascinating study by Olivia Zahn et al which uses deep learning neuronal networks (DNN) that model the hovering flight of moths (Manduca sexta) in order to explore how pruning of a fully connected network affects the performance of the network model. The authors do a great job in introducing and discussing the role of synaptic pruning in the development of neuronal networks as has been shown in many different biological systems such as the classic Hubel Wiesel studies of the visual cortex. This broad discussion makes this study interesting for a general readership. In their study the authors train a fully connected network to generate hovering with a minimum error. Subsequently they used manual and computational approaches to prune these fully connected DNNs to different levels of sparsity to find the optimal sparse network configuration, which is still capable of controlling moth hovering. Perhaps, unexpected to the authors (but not unexpected to this reviewer) the authors find that their networks perform better than the median performance of the fully connected networks when pruned up to 85% sparsity. Indeed, of the 1320 DNNs that they trained 858 were optimally sparse at 93% sparsity. It was also surprising that some parameters, such as the initial head-thorax angular velocity, were completely pruned out as it had no impact on the output and predictive power of the network. The authors also found that these pruned networks exhibit sharp performance limits when pruned beyond 93%. This was found at all layers of the networks. Of course, one major caveat is that the biological reality is more rich, and complex than the forward model networks that were generated to simulate moth hovering. But, to this reviewer, this is a very minor issue that does not detract from the fundamental lessons learned from this study: i.e. that sparsity imbues neuronal network with increased capabilities. Many popular, contemporary, computational models of rhythmogenic networks are fully connected ball-and-stick models which are inspired e.g. by the very small network of the stomatogastric ganglion. However, in reality, fully connected networks are an exception and most networks are not fully connected but exceedingly sparse. With the sparsity comes e.g. a characteristic cycle-by-cycle variability that is often ignored. The

authors have done a fabulous job in discussing the universal role of pruning in their discussion, but I highly recommend that the authors also discuss the existence of sparsely connected networks in biology. This is currently missing in this manuscript. One such network is the mammalian respiratory network which is sparsely connected. This network has to be extremely flexible, yet also very robust. It is characterized by a high cycle variability, and sparse connectivity. The cortex is also sparsely connected. Indeed, because of the sparse connectivity of mammalian neuronal networks, so little is known about the circuit diagrams underlying mammalian neuronal networks: it is exceedingly difficult to reproducibly find connected pairs of neurons. Because of the sparsity no mammalian network circuit exists that looks like the STG or the leech heartbeat system. Thus, I highly recommend that the authors discuss the presence of sparsely connected network which will provide further validity for the fundamental conclusions drawn in this very inspiring computational study.

Thank you for the suggestion to discuss sparsely connected networks in biological systems. We agree that this is an interesting and relevant connection and have thus added a discussion of sparse biological networks to our **Introduction in paragraph 4**. Here is the added text:

"In fact, a diversity of sparse networks exist across species. For example, the respiratory rhythm patterns of mammals are generated by sparsely connected networks [23]. In the olfactory system of *Drosophila*, high-dimensional odor signals are sparsely encoded via the mushroom body [24, 25]. Neural network pruning enables the exploration of biologically-inspired, sparse learning and the strengths of the resultant sparse networks."

Changes to manuscript

1. Replaced *"overparameterized"* with *"over-parameterized"* in p1 of Introduction.
2. Added text: *"The biological mechanisms that underlie synaptic pruning (often activity dependent) have a range of processes including a variety of semaphorins, increased GABAergic signaling, changes in dendritic spine density (with some enigmatic mechanisms), and even neuro-immune interactions [6]."* in p1 of Introduction.
3. Added text: *"In fact, a diversity of sparse networks exist across species. For example, the respiratory rhythm patterns of mammals are generated by sparsely connected networks [23]. In the olfactory system of Drosophila, high-dimensional odor signals are sparsely encoded via the mushroom body [24, 25]. Neural network pruning enables the exploration of biologically-inspired, sparse learning and the strengths of the resultant sparse networks."* in p4 of Introduction.
4. Replaced *"fully connected"* with *"fully-connected"* in p6 of Introduction.
5. Replaced *"and Tables S1 and S2"* with *"S6 Table, and S7 Table"* in p1 under Moth Model of Materials and methods.
6. Replaced *"magnitude of force applied"* with *"average force applied by the wings during each downstroke and upstroke"* in p2 under Moth Model of Materials and methods.
7. Added text: *"In addition to the downstroke or upstroke averaged forces, the model includes gravitational forces, abdominal torques, and drag forces on the body."* in p2 under Moth Model of Materials and methods.

8. Added text: *"Thus our model is basically a simplified two-body dynamical system with thrust vectoring. Since our dominant focus is on hovering flight, this provides a reasonable basis for examining the control consequences of pruning a deep neural network. Fig. 6 shows three example hovering trajectories of the simulated insect. All trajectories begin at the origin ((x,y) = (0,0)). The grey dotted lines show the trajectory of the center of mass of each body segment and the red dotted line shows the trajectory of the thorax-abdomen joint."* in p2 under Moth Model of Materials and methods.
9. Replaced *"Table 4"* with *"S8 Table"* in p1 under Generating training data of Materials and methods (2x).
10. Added second power in Equation 1.
11. Replaced *"Table S1"* with *"S6 Table"* in p3 of Discussion.
12. Replaced *"overparameterized"* with *"over-parameterized"* in p5 of Discussion.
13. Added text: *"The results of this study pose an interesting question about how the size of the initial network architecture affects the resultant pruning statistics. The networks pruned in this study are feed-forward, each with four hidden layers with 400, 400, 400, 16 nodes, respectively. This choice of architecture is somewhat arbitrary, however through the process of tuning and cross-validating the fully-connected network, we converged to a set of hyperparameters (including the size of the hidden layers) which resulted in the most optimally performing network. To begin to explore the effect that initial network architecture size has on the pruning statistics, we repeated the experiment with increasingly smaller network architectures. For example, in S1 Fig we trained 400 networks, each with four hidden layers with 200, 200, 200, 8 nodes. S2 Fig and S3 Fig show the same results for networks of sizes 100, 100, 100, 8 and 50, 50, 50, 8, respectively. These decreases in hidden layer widths correspond to a decrease in the total number of weights across the networks from 330, 512 (for the original networks in Fig. 4) to 83, 656 (S1 Fig), 21, 856 (S2 Fig), and 5, 956 (S3 Fig). Across all networks, there is a slight improvement in performance for low levels of pruning. All networks show a performance breakdown, however the sparsity at which the breakdown occurs changes with the size of the network. For example, the networks in S3 Fig show a performance breakdown at 65% or when there are 2, 084 weights remaining. This is compared to the original 1320 networks which showed a performance breakdown at 93% or when there are 23, 135 weights remaining. The initial architecture of the network affects the achievable sparsity by the pruning protocol employed here. Additionally, smaller network architectures result in more volatility when higher levels of sparsity are reached. However, the results of these preliminary experiments only begin to explore the relationship between initial network architecture and resultant pruning statistics. We found that as the network architecture is made smaller, the raw number of parameters post-pruning is fewer. Whether these extra small networks are as robust to noise or better at generalizing to unseen data is yet to be seen. It is also unclear what the optimal starting architecture size should be because large, over-parameterized networks are thought to be more efficient to optimize via gradient descent [44]. As stated, these preliminary experiments open up many interesting questions to be explored in future work."* to p8 of Discussion.
14. Replaced *"University of Washington Data Science Grant from the Moore Foundation, Sloan Foundation , and the Washington Research Foundation"* with *"Washington Research Foundation and by a Data Science Environments project award from the Gordon and Betty Moore*

Foundation (Award #2013-10-29) and the Alfred P. Sloan Foundation (Award #3835) to the University of Washington eScience Institute" in Acknowledgements.

15. Added Supporting figures 1-3.
16. Reformatted Supporting Information.
